# Enhancing Natural Killer Cell Targeting of Pediatric Sarcoma

**DOI:** 10.3389/fimmu.2021.791206

**Published:** 2021-11-04

**Authors:** Natacha Omer, Wayne Nicholls, Bronte Ruegg, Fernando Souza-Fonseca-Guimaraes, Gustavo Rodrigues Rossi

**Affiliations:** ^1^ The University of Queensland Diamantina Institute (UQDI), The University of Queensland, Brisbane, QLD, Australia; ^2^ Oncology Services Group, Queensland Children’s Hospital, South Brisbane, QLD, Australia; ^3^ Faculty of Medicine, The University of Queensland, Brisbane, QLD, Australia

**Keywords:** osteosarcoma, Ewing sarcoma, rhabdomyosarcoma, natural killer, immunotherapy

## Abstract

Osteosarcoma, Ewing sarcoma (EWS), and rhabdomyosarcoma (RMS) are the most common pediatric sarcomas. Conventional therapy for these sarcomas comprises neoadjuvant and adjuvant chemotherapy, surgical resection of the primary tumor and/or radiation therapy. Patients with metastatic, relapsed, or refractory tumors have a dismal prognosis due to resistance to these conventional therapies. Therefore, innovative therapeutic interventions, such as immunotherapy, are urgently needed. Recently, cancer research has focused attention on natural killer (NK) cells due their innate ability to recognize and kill tumor cells. Osteosarcoma, EWS and RMS, are known to be sensitive to NK cell cytotoxicity *in vitro*. In the clinical setting however, NK cell cytotoxicity against sarcoma cells has been mainly studied in the context of allogeneic stem cell transplantation, where a rapid immune reconstitution of NK cells plays a key role in the control of the disease, known as graft-versus-tumor effect. In this review, we discuss the evidence for the current and future strategies to enhance the NK cell-versus-pediatric sarcoma effect, with a clinical focus. The different approaches encompass enhancing antibody-dependent NK cell cytotoxicity, counteracting the NK cell mechanisms of self-tolerance, and developing adoptive NK cell therapy including chimeric antigen receptor-expressing NK cells.

## Introduction

Osteosarcoma, Ewing sarcoma (EWS), and rhabdomyosarcoma (RMS) are the most common sarcomas in children. Patients with metastatic, relapsed, or refractory pediatric sarcoma have a dismal prognosis with less than 30% maintaining long-term survival (1). Further, no significant improvement in patient outcome has been made over the last 3 decades with currently available therapies (surgery, radiation, and chemotherapy) (2–4). Hence, there is an urgent need for innovative therapeutic interventions, such as immunotherapy.

**Figure 1:**
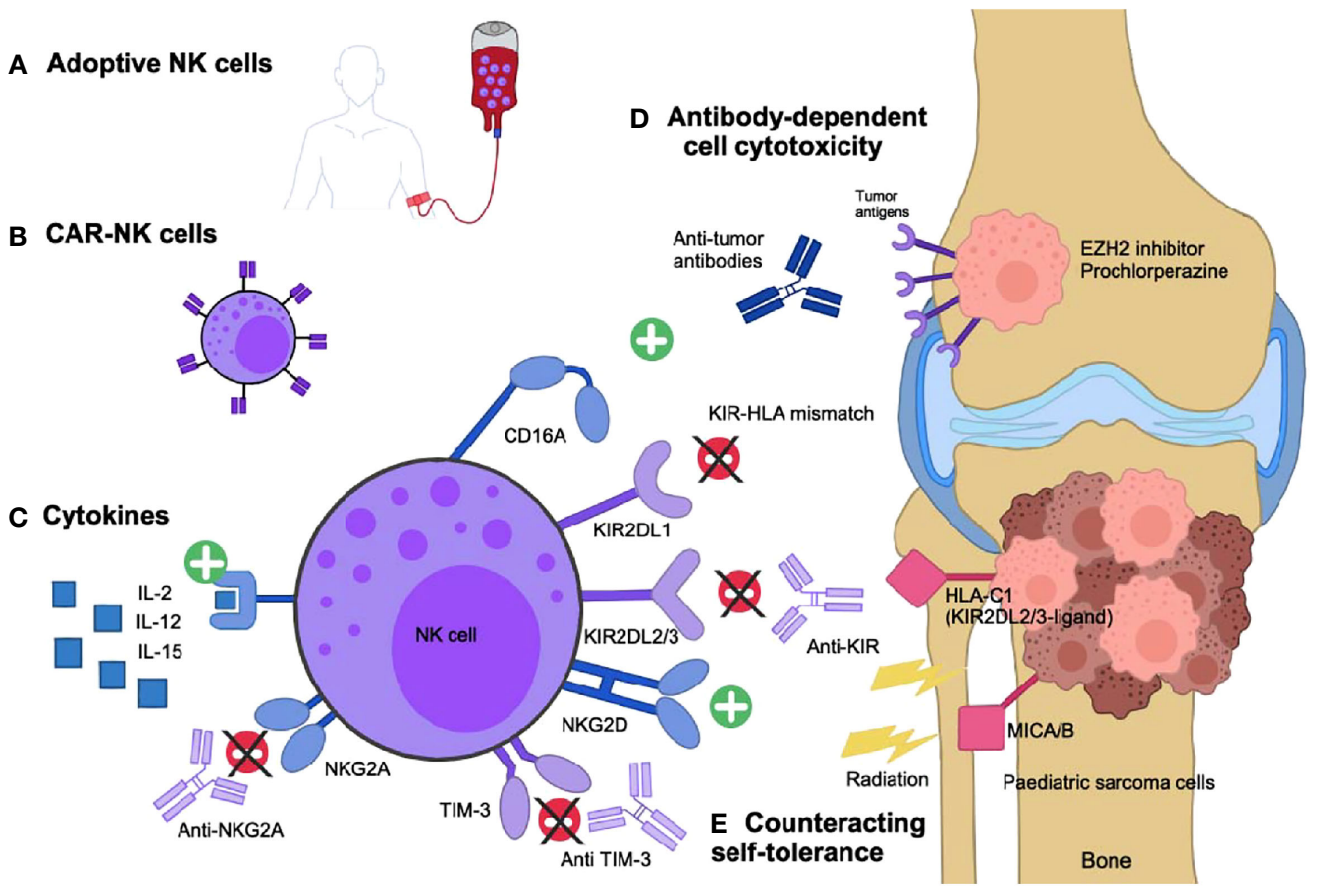
Enhancing NK cells-versus-pediatric sarcoma effect. **(A)** Expanded and activated NK-cells *in vitro* or **(B)** engineered NK cells expressing combined antigen-receptors (CAR) specific to the sarcoma cells can be infused to the patients. **(C)** Infused cytokines can stimulate the NK cell cytotoxic activity. **(D)** Specific anti-tumor antibodies activate NK cells *via* their CD16 activating receptor and drive the antibody-dependent cell cytotoxicity (ADCC). Small molecules, such as EZH2 inhibitors and prochlorperazine, can increase antigen presentation to facilitate ADCC. **(E)** Self-tolerance can be counteracted if the NK cells are KIR-mismatched to the tumor, if the NK inhibitory receptors are targeted with specific inhibitory antibodies (anti-KIR, NKG2A or TIM-3) or if the NK activating receptors ligands, such as MIC-A/B, are overexpressed due to genotoxic stress such as radiation therapy.

Immunotherapy is not a new concept for sarcoma. The earliest described sarcoma immunotherapy was in 1891, when William B. Coley observed tumor regression after locally injecting *Streptococcus* bacteria into patient’s sarcoma to generate an immune response (5). Now scientific and clinical evidence strongly supports the critical role for both early and late immune responses to control cancer growth. As an example, there is mounting evidence that hematopoietic stem cell transplantation (HSCT) has an allograft-versus-tumor effect in the treatment of leukemia and in a subgroup of solid tumors including sarcomas (6). Rapid immune reconstitution post HSCT is critical for the anti-tumor effect, particularly due to the recovery of natural killer (NK) cells (7). Nevertheless, studies investigating the function of NK cells in sarcoma in the clinical field are limited and further work aiming to “unleash” the full potential of NK cells to improve their anti-sarcoma activity are needed. This review highlights the current knowledge in the field and future perspectives of applying NK cell-based immunotherapies to treat pediatric sarcomas. Possible strategies to increase NK cells efficiency discussed here are the use of monoclonal antibodies (mAb) targeting tumor antigens or of pharmacological agents to enhance antibody-dependent cell-mediated cytotoxicity (ADCC), the use of cytokines to enhance NK cell-mediated anti-tumor activity, strategies to counteract self-tolerance, and the development of adoptive therapy using NK cells, including chimeric antigen receptor (CAR)-expressing NK cells (**Figure 1**).

## NK Cell Activity in Pediatric Sarcoma

NK cells are cytotoxic innate immune cells that can eliminate infected or transformed cells without prior sensitization. NK cells express inhibitory surface receptors, such as killer-cell immunoglobulin receptors (KIR), which recognizes specific human leucocyte antigen (HLA) class I molecules HLA-A, B and C, and CD94/NK group 2 member A (NKG2A), which recognizes HLA-E (8). NK cells are educated to lyse target cells lacking expression of major histocompatibility complex (MHC) class I molecules expressed by the host cells (9). Their activating surface receptors include natural cytotoxic receptors (NCRs) and NK group 2 member D (NKG2D), which recognize stress proteins on the surface of target cells such as MICA/B and ULBPs, and a Fcγ receptor CD16, that mediate ADCC through recognition of the Fc portion of antibodies on opsonized cells (10). Coreceptors of NCRs and NKG2D, such as DNAM-1, are capable of amplifying the NK cell activation (11). The balance between activating and inhibitory signals received by the NK cells determines their cytotoxic activity. This activity is mediated by the release of cytotoxic granules containing granzymes and perforin, expression of death receptor ligands on their surface, and the production of cytokines (e.g., tumor necrosis factor-α and interferon γ) promoting an anti-tumor immune response (12).

In pre-clinical studies, osteosarcoma, EWS, and RMS cells are sensitive to killing by NK cells. NK cells from healthy donors expanded for 7 days with K562-mb15-41BBL feeder cells showed a median cytotoxicity of 87.2%, 79.1% and 46.1% at a 1:1 effector:target ratio with EWS, RMS and osteosarcoma cells respectively. EWS cells were particularly sensitive with maintained cytotoxicity at considerably lower ratios (13). Cytotoxicity was not related to levels of expression of NK receptor ligands but was markedly inhibited by preincubation of NK cells with antibodies anti-NKG2D and anti-DNAM-1, when used in combination (13). Similarly, NKG2D receptor blockade, but not that of DNAM-1, significantly decreased NK cells cytotoxicity *in vitro* against osteosarcoma cells (14). KIRs were also shown to play an important role: KIR receptor-ligand mismatched NK cells showed higher cytotoxicity *in vitro* against osteosarcoma cells, and this was enhanced further when osteosarcoma cells HLA class I molecules were blocked (14). Furthermore, in a mouse xenograft model of EWS, weekly intravenous administration of expanded NK cells decreased significantly the number of lung metastases (15). In an orthotopic xenograft mouse model of osteosarcoma, intra-tumoral injection of *in vitro* activated and expanded NK cells and intraperitoneal IL-2 for 5 days limited bone damage and tumor growth, prevented lung metastases, and significantly prolonged mice survival (14). *Ex vivo* expanded NK cells with IL-15 and IL-21 injected after radiation therapy to mice with subcutaneous RMS slowed the tumor growth significantly (16). In another xenograft model of RMS, adoptive therapy of NK cells completely prevented the intraperitoneal implantation of RH30 tumor cells (17).

In the clinical setting, however, the specific NK-versus-sarcoma effect has only been studied in small case series. In one study, two patients with stage 4 EWS were in complete remission after haploidentical transplantation. Early post-transplant, their rapidly recovering NK cells demonstrated high cytotoxic activity against EWS cell lines *in vitro*, suggesting a potential role in systemic tumor control (18). In another study, one patient with relapsed metastatic RMS and one with EWS responded to a haploidentical transplantation after 3 lines of chemotherapy. The patient with RMS had a full donor NK genotype at 18 months post-transplantation and his NK cells exhibited high lysis of K562 cells, a classical target of NK cells due to their lack of HLA class I and II expression (19). These reports suggest that if the NK cells effector functions are maximized, clearance of high-risk sarcoma can be achieved.

## Therapeutic Enhancement of NK Cell Functions *via* ADCC

NK cells are the ideal candidate for adoptive therapy combined with mAbs targeting specific tumor antigens due to their unique mechanism of target cell lysis through ADCC mediated by their CD16 (FcγRIIIa) receptors (20). Multiple preclinical studies have shown the benefit of NK cells and mAbs in pediatric sarcomas (21–23). Several mAbs have been tested as single agent in phase I or II clinical trials, mainly in patients with osteosarcoma, including trastuzumab to target human epidermal growth factor receptor 2 (HER2) (24), cexutimab to target the epidermal growth factor receptor (EGFR) (25), glembatumumab-vedotin to target the glycoprotein non-metastatic B (GPNMB) (26), and dinutuxumab to target disialoganglioside GD2 (27). Cixutumumab, ganitumab, robatumumab, and figitumumab, targeting the insulin-like growth factor-1 receptor (IGF1R), have been tested in advanced RMS, EWS, and osteosarcoma (28, 29). Bevacizumab has been used to target vascular endothelial growth factor (VEGF) in RMS (30). Unfortunately, none of these clinical trials have shown a significant clinical benefit. Of note, most included patients with the tumor type of interest without restriction. Inclusion criteria based on the presence of predictive biomarkers identifying patients who would likely respond to these mAb are needed to determine the utility of these agents. The clinical response to mAb is also affected by a single nucleotide polymorphism in the FCGR3A (CD16) gene that affects binding affinity for IgG (increased affinity with valine at FCGR3A-158) (20).

Strategies to improve mAbs interactions with CD16 have been developed in other tumor types. Obinutuzumab, a humanized Fc-defucosylated anti-CD20 mAb, has an increased CD16 binding affinity, insensitive to CD16-V158F polymorphism (31). Bispecific or trispecific killer engagers have been generated and are composed of anti-CD16 Ab connected to the scFv of one or two of the tumor-antigen antibodies (31).

The ADCC can also be amplified by upregulating the expression of targeted surface antigens or by limiting their endocytosis. Kailayangari et al. demonstrated that the use of an enhancer of zeste homolog 2 (EZH2) inhibitor enhanced the expression of GD2 on the surface of EWS sarcoma cells, thus increasing their sensitivity to lysis by GD2 specific CAR-T cells (32). Prochlorperazine, used as anti-psychotic and anti-emetic agent in the clinic, reversibly inhibits dynamin-dependent endocytosis and, by enhancing target availability, improved the efficiency of NK cell-mediated ADCC by cetuximab, trastuzumab and avelumab in non-sarcoma solid tumor models (33).

Even though the mAbs bind to the activating receptor CD16 on NK cells, a potent activating signal, they have not shown positive results in pediatric sarcoma (20). Combination with adoptive NK cell therapy, small molecules or other strategies to amplify ADCC, as well as a better selection of patients to include in trials based on their tumor expression profile, could improve the mAbs efficacy.

## Maximize Effector Functions With Cytokines

Cytokines have raised interest as an adjuvant therapy in sarcomas since the 1980s. Mice treated with interferon (IFN)-α, IL-2, IL-12, IL-15, or IL-18 showed an increase in NK cell cytotoxicity against multiple sarcoma cell subtypes, but few have been translated successfully to the clinic (34). Pegylated IFN-2b in addition to conventional chemotherapy was tested in osteosarcoma in an international phase 3 clinical trial (35). Unfortunately, it showed no benefit as a maintenance therapy. This study had some limitations: approximately 45% of the patients did not start or complete the treatment with pegylated IFN-2b due to the intensity of treatment, patient’s refusal, physician choice or toxicity (35). Similarly, IL-2 therapy is limited by its severe side-effects when used systematically (36). When administered by aerosol in combination with NK cell infusion in a mouse model of metastatic pulmonary osteosarcoma, the NK cell number in the lungs was increased and IL-2 induced metastatic regression and improved overall survival of the animals (37). There is an ongoing clinical trial in metastatic osteosarcoma using aerosol IL-2 (NCT01590069). IL-15 was tested in a phase I clinical trial as a 10-day continuous infusion in 27 adults with advanced metastatic solid tumors, including 4 sarcomas (38). The maximum tolerated dose was reached, and 8 serious adverse events were reported including 2 deaths. A dramatic increase in NK cell number was induced: 38-fold in total circulating NK cells and 358-fold in the highly inflammatory CD56^bright^ NK cell subset.

These reports demonstrate a clear activity of cytokines in boosting NK cell function in sarcoma, but the potential for toxicity make them difficult to apply in the clinic. The use of NK cells expanded and stimulated with cytokines before their therapeutic infusion, or the use of genetically modified NK cells may help overcome these limitations (12).

## Tipping the Balance Toward Activation

There is a fine balance between activating and inhibitory signals regulating NK cell cytotoxicity and self-tolerance, and the tumor microenvironment (TME) of solid tumors is known to be immunosuppressive (39). A detailed characterization of the immune TME in sarcomas is lacking and it is currently unclear which approach should be applied to which type of sarcoma to target the TME appropriately (40). Proinflammatory cytokines, as discussed above, is a possible method. Immune checkpoint inhibitors reversing the exhausted phenotype of T cells are another strategy (12). However, the role of well-described T cell immune checkpoints such as PD-1, CTLA-4, TIM-3, and B7-H3 in the control of NK cell tolerance is unclear and they seem to promote different effects than those described and expected for T cells (41). Nevertheless, TIM-3 was found to be expressed on NK cells, and anti-TIM-3 antibody in combination with a superagonist of IL-15 (ALT-803) enhanced NK cell cytotoxicity against osteosarcoma cells *ex vivo* (42). Further, specific antibodies to block NK cell immune checkpoints have recently been developed and are currently undergoing trials in solid tumors, such as the anti-KIR mAb, lirilumab (NCT02813135), and the anti-NKG2A antibody, monalizumab (NCT02671435).

An alternative approach to break the self-tolerance and enhance NK cell anti-tumor activity is through their activating receptors. The ligands of the activating receptor NKG2D are upregulated following genotoxic stress such as viral infection or radiation therapy, alerting the immune system to potentially dangerous transformed or infected cells (12). The combination of NK cell and radiation therapy has shown promising results. Dogs with osteosarcoma die of metastatic progression to the lungs in 80% of the cases, but in a canine clinical trial, local radiation therapy before intralesional NK cell injection significantly increased NK cell homing to the tumor and, encouragingly, 50% of the dogs were metastasis-free 6 months after NK transfer (43).

Another method to avoid the inhibition of NK cells is the use of KIR-HLA mismatched cells. The interaction of KIR receptors on NK cells with their cognate HLA ligands provides a strong inhibitory signal preventing NK-mediated lysis of the self-target cells (8). Therefore, low MHC-I-expressing tumors are an ideal target for NK cells. MHC I downregulation or absence of expression has been reported in high-risk EWS and RMS (44). Osteosarcoma cells with surface expression of HLA molecules are less susceptible to killing by NK cells compared to cells lacking this expression (45). Similarly, KIR-HLA mismatching (i.e., donor cells expressing KIRs incompatible with recipient HLA ligands) can lessen the inhibitory signals from the sarcoma cells received by NK cells and result in enhanced anti-tumor function. Osteosarcoma cells target killing correlate with their degree of KIR-HLA incompatibility with the NK cells (45). Clinical data also demonstrate that KIR-mismatched NK cells exert enhanced antitumor activity in patients with solid tumors undergoing allogeneic HSCT and even in patients undergoing autologous HSCT (46, 47). A Japanese group has successfully developed KIR-mismatched cord-blood cells transplantation with reduced-intensity conditioning as a form of non-targeted immunotherapy (as the anti-GD2 antibodies are not approved by the regulatory authorities in Japan) to produce excellent outcomes in patients with high-risk neuroblastoma (48). KIR-mismatched stem cell transplantations have not been tested specifically in patients with pediatric sarcomas, but better overall responses were observed in patients who had undergone HLA-haploidentical stem cell transplantation with 1 to 2 KIR-HLA mismatches, when retrospectively studied (6, 46). Although not targeting sarcoma, anti-CD19 CAR-NK cells developed recently for relapsed or refractory anti-CD19-positive cancers have shown great clinical responses when produced from selected KIR-HLA mismatched cord-blood units (49).

These examples demonstrate the importance of breaking NK cell tolerance to enhance NK cell-versus-sarcoma activity, not only in the context of allogeneic stem cell transplant, but also of adoptive NK cell therapy.

## Adoptive Therapy

NK cells for adoptive therapy can be derived from multiple autologous or allogeneic sources including peripheral blood, umbilical cord blood, CD34^+^ hematopoietic progenitors, and human-induced pluripotent stem cells (12).

Multiple trials have tested adoptive NK cell (aNK) transfer post HSCT in high-risk sarcomas with the intent to amplify the graft-versus-tumor effect. In 2015, Shah et al. trialed donor-derived NK cell transfer in 9 children and young adults with high-risk solid tumors. The NK cells were activated *in vitro* with IL-15 and 4-1BBL and transferred following HLA-matched T cell depleted HSCT. Five patients with relapsed EWS and 1 with RMS were enrolled, and 3 of these 6 patients survived over 2 years, including 2 in complete remission. Interestingly, 5 of 9 transplant recipients experienced graft-versus-host disease following the NK cell infusion (50). Perez-Martinez et al. stimulated NK cells with IL-15 and infused them 30 days after haploidentical HSCT in 6 patients with high-risk solid tumors, including 3 EWS and 1 osteosarcoma, all with progressive disease (51). Four patients showed a clinical response (3 patients in partial remission of their tumor and 1 with stable disease). No toxicity secondary to the NK cell infusion was reported. Thakar et al. also tested adoptive transfer of donor NK cells post haploidentical HSCT in 14 patients with relapsed pediatric sarcomas (9 EWS, 4 RMS and 1 osteosarcoma) with stable disease, and demonstrated much better than expected survival in this high-risk population with an overall survival of 64% and 40% at 1 and 2 years respectively (75% and 45% for the EWS cohort) (52).

Outside of the post-HSCT context, aNK cells are only recently being tested in sarcoma with only 8 clinical trials ongoing, or recently completed (**Table 1**). Most of these trials are testing NK cell infusions in combination with a cytokine (IL-15 or IL-2) after chemotherapy conditioning with at least cyclophosphamide. The NK cells used are expanded from autologous or heterologous sources including universal donors, cord blood, or parental donors (haploidentical).

**Table 1:** Clinical trials testing adoptive NK cell therapy in sarcoma.

Clinicaltrial.gov identifier	Tumor type included	NK cell type	Phase	Location	Methods	Status/Publication
NCT02890758	Relapsed/refractory soft tissue sarcoma (EWS and RMS included)	Universal donor	I	USA	NK cell infusions (x2) +/- recombinant IL-15 - Dose escalation with 3 cell doses	Active, Not recruiting
NCT03420963	Relapsed/refractory solid tumor	Cord blood-derived expanded	I	USA	NK cell infusion (x1) on D8 - Conditioning with cyclophosphamide and etoposide (D1 to 5)	Recruiting
NCT03941262	Refractory cancer	*Ex-vivo* expanded autologous	I	USA	NK cell infusion weekly for 5 weeks +/- Avelumab or Pembrolizumab (anti-PD-L1 and anti-PD-1) - Dose escalation with 3 cell doses	Recruiting (53, ASCO abstract)
NCT04214730	Advanced solid tumor	*N/A*	?	China	NK cell infusion (x4) + chemotherapy vs chemotherapy only	Recruiting
NCT01875601	Relapsed/refractory solid tumor	Autologous activated	I	USA	NK cell infusion (x1) - Conditioning with cyclophosphamide +/- recombinant IL-15 - Dose escalation cell dose and IL-15	Completed (unpublished)
NCT02849366	Recurrent sarcoma	*N/A*	I/II	China	Cryosurgery +/- NK cells infusions (x3)	Completed (unpublished)
NCT03209869	Relapsed/refractory neuroblastoma and osteosarcoma	*Ex-vivo* expanded activated haploidentical	I	USA	NK cell infusion + humanized 14.18-IL2 (anti-GD2 immunocytokine) (D1 to 7)	Suspended (due to COVID-19)
NCT02409576	EWS, RMS	*Ex-vivo* expanded activated haploidentical	I/II	Singapore	NK cell infusion (x1) on D0 - Conditioning with cyclophosphamide, fludarabine and radiation 2Gy + IL-2	Unknown

This table outlines completed or ongoing clinical trials of NK cell adoptive therapy including patients with osteosarcoma, Ewing sarcoma or rhabdomyosarcoma. Underlined clinical trials include children. The aims of these clinical studies are to determine safety (phase I) and/or efficacy (phase II) of the interventional therapies. Most involve a conditioning with chemotherapy +/- radiation therapy before infusion of the cells (number of cell infusion planned in bracket), and cytokine injections to enhance the cell activity (IL-2 or IL-15). EWS, Ewing sarcoma; RMS, rhabdomyosarcoma; NK, natural killer; N/A, non available; Gy, Gray; D, Day.

In a preliminary report, Chawla et al. tested activated autologous NK cells (SNK01) in a dose-escalation phase 1 study in patients with rapidly progressive metastatic solid tumors (53). Of the 7 patients enrolled so far, 5 had sarcomas and had received an average of 5 prior lines of therapy. The best overall response at 9 weeks was stable disease in 4 patients. No adverse events were reported and dose limiting toxicity had not been reached, but dose escalation is ongoing.

## CAR-NK Cells

CAR-NK cells are harder to engineer than CAR-T cells, but they are better tolerated and can be readily available as an allogeneic off-the-shelf product (54). CAR-NK cells maintain their ability to be activated by their innate receptors recognizing transformed cells, without prior antigen priming needed, while antigen recognition is redirected toward CAR-specific targets. Furthermore, CAR-NK cells can respond to levels of tumor-associated antigens that are too low to trigger ADCC (12).

In a phase I/II clinical trial of cord blood-derived CAR-NK cells treating patients with relapsed/refractory B-cell leukemia/lymphoma, the overall response rate was 73% including 64% with complete response (49). In contrast to large trials of CD19‐directed CAR T‐cell therapy with a comparable response rate of up to 80% (55), none of the patients showed evidence of neurotoxicity or cytokine-release syndrome. CAR‐NK cells persisted up to 12 months after infusion.

In pediatric sarcoma, CAR-NK cells have shown anti-cancer activity *in vitro.* GD2-specific CAR-NK had an increased activity against EWS cells in an antigen-specific manner, but when transferred to mice with GD2-positive EWS xenografts they lacked efficiency due to inhibitory HLA-G hyper-expression on the tumor (56). Anti-receptor tyrosine kinase-like orphan receptor 1 (ROR1) CAR-NK cells also demonstrated significantly enhanced cytotoxicity *in vitro* against EWS and osteosarcoma cell lines compared to mock expanded NK cells (57). However, to date, there is no clinical trial of CAR-NK in sarcoma registered on clinicaltrial.gov.

Overall pre-clinical data in sarcoma and clinical trials of CAR-NK cells in other cancers show how promising CAR-NK cells could be as an immunotherapeutic approach in pediatric sarcomas.

## Conclusion

NK cells have recently emerged as an exciting option to target sarcomas resistant to conventional anti-cancer therapy. Many immunotherapies use NK cells as one of the main effectors of their anti-cancer effect along with T cells, but this effect can be enhanced, and adoptive NK cell therapy and particularly CAR-NK cells are emerging from preclinical settings. Ongoing research appears promising to translate this into patient’s benefit in the coming years.

## Author Contributions

Writing – original draft preparation, NO. Writing – review and editing, NO, WN, BR FS-F-G and GR. Figure creation - NO and BR. All authors contributed to the article and approved the submitted version.

## Funding

NO is supported by the Queensland Children’s Hospital and UQ PhD scholarship. FS-F-G is funded by a UQ Diamantina Institute laboratory start-up package, Australian and New Zealand Sarcoma Association Sarcoma Research Grant, a priority-driven collaborative cancer research scheme grant co-funded by Cancer Australia and Cure Cancer (#1158085), and a US Department of Defense – Breast Cancer Research Program – breakthrough award level 1 (#BC200025).

## Conflict of Interest

FS-F-G is a consultant and has a funded research agreement with Biotheus Inc. 

The remaining authors declare that the research was conducted in the absence of any commercial or financial relationships that could be construed as a potential conflict of interest.

## Publisher’s Note

All claims expressed in this article are solely those of the authors and do not necessarily represent those of their affiliated organizations, or those of the publisher, the editors and the reviewers. Any product that may be evaluated in this article, or claim that may be made by its manufacturer, is not guaranteed or endorsed by the publisher.
